# Prophylactic placement of a covered nitinol stent to prevent carotid blowout in a patient with supraclavicular lymph node metastasis from esophageal cancer

**DOI:** 10.1186/s40064-015-1243-9

**Published:** 2015-08-25

**Authors:** Takeshi Fujita, Katsuyoshi Ito, Masahiro Tanabe, Naofumi Matsunaga

**Affiliations:** Department of Radiology, Ube Industries, Ltd. Central Hospital, 750 Nishikiwa, Ube, Yamaguchi 755-0151 Japan; Department of Radiology, Kawasaki Medical School, 577 Matsushima, Kurashiki, Okayama 701-0192 Japan; Department of Radiology, Yamaguchi University Graduate School of Medicine, 1-1-1 Minamikogushi, Ube, Yamaguchi 755-8505 Japan

**Keywords:** Prophylactic, Covered nitinol stent, Carotid blowout, Lymph node metastasis

## Abstract

Enlargement of primary tumor and metastatic lymph nodes in patients with head and neck cancer can be progressive and invade the surrounding vessels despite intensive treatment. Carotid blowout (CBS) tends to occur in these patients, and prompt treatment is required. Surgical management of carotid blowout is technically troublesome because exploration and repair of the previously irradiated or tumor-invaded field are difficult. Endovascular therapy with stent deployment is a good alternative to surgery. Even with such interventional procedures as stent grafting, it is sometimes difficult to obtain favorable outcomes in end-stage patients with poor general conditions. The prophylactic placement of a covered nitinol stent was performed to prevent carotid blowout in a patient with supraclavicular lymph node metastasis from esophageal cancer, and fatal bleeding due to carotid blowout was avoided. The usefulness of the prophylactic placement of a covered nitinol stent for preventing carotid blowout in an end-stage patient is presented.

## Background

Patients with esophageal cancer have a poor prognosis because they often have no symptoms until their disease is advanced (Schweigert et al. 2013). In most patients with advanced cancer, mediastinal, supraclavicular, and intra-abdominal lymph node metastases occur (Schweigert et al. 2013; Schoppmann et al. 2013). Irradiation and systemic chemotherapy are the mainstay for treating these patients (Meng et al. 2013). Despite intensive treatment, progressive enlargement of primary tumor and metastatic lymph nodes can occur. This can result in regional tumor invasion to the surrounding structures, which is frequently associated with airway obstruction, a tracheo-esophageal fistula, or rupture of large arteries (Pennathur et al. 2013).

The mortality and morbidity rates associated with carotid rupture are reported to be unacceptably high (Chaloupka et al. 1999). The treatment is usually surgical exploration with ligation of the involved vessel; however, infections, radiation-induced necrosis, and prior surgical exploration make the surgical approach technically difficult (Katras et al. 1999).

Interventional procedures, including percutaneous stent placement or coil embolization, provide a better alternative to surgery, accompanied by less patient morbidity and suffering (Pyun et al. 2007; Chang et al. 2007).

Prophylactic placement of a covered nitinol stent to prevent carotid rupture in a patient in whom the metastatic lymph node invaded directly to the surrounding common carotid artery is reported.

## Case presentation

A 62-year-old man, who had known T3, N4, M0, Stage IVa squamous cell carcinoma of the middle esophagus, presented with dysphagia and was admitted to our institute. Regional radiotherapy extending from the supraclavicular fossa to the pericardial area, including the entire mediastinum, was planned. A total dose of 50.4 Gy of radiation therapy was delivered and systemic chemotherapy consisting of low-dose cisplatin and 5FU was concurrently performed. Eight months after the initial treatment, right supraclavicular lymph node enlargement was seen on follow-up computed tomography (CT). Although 20 Gy of additional irradiation were given to the right supraclavicular fossa, the metastatic lymph node enlarged and invaded the surrounding soft tissue. Left common carotid artery was confirmed to be intact on contrast enhanced CT.

**Fig. 1:**
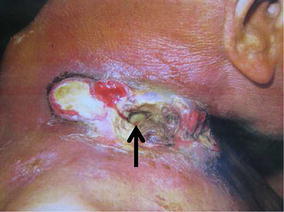
Photograph of the affected area. The metastatic lymph node accompanied with necrotic tissue after irradiation in the right supraclavicular region destructively invades the surrounding cervical area of the patient. The right carotid sheath and arterial pulsation are confirmed in the cutaneous pocket under direct vision (*arrow*)

Inspection of the neck confirmed the presence of a large mass in the right supraclavicular region with carotid sheath invasion. Necrotic skin caused by the irradiation was also observed within the tumor. As a result, direct pulsation of the common carotid artery was visualized in the cutaneous pocket on gross examination (Fig. 1).

**Fig. 2:**
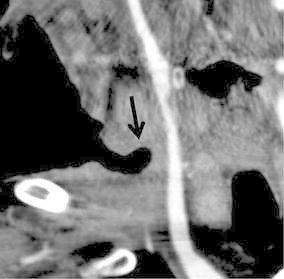
Multiplanar reconstruction contrast-enhanced computed tomography (CT) in the coronal view shows the tumor invading to the right cervical region of the patient, and a large cutaneous pocket adjacent to the right carotid artery is revealed (*arrow*)

It was determined that the patient’s condition was critical, and the decision was made to proceed with a percutaneous interventional approach (Fig. 2). It was decided that a covered nitinol stent graft could be placed to prevent rupture of the right carotid artery.

After obtaining written, informed consent from the patient, an 8-French (8-Fr) angiographic sheath was placed via the right femoral artery. Radiopaque markers were placed on the right neck surface as markers of the appropriate position planned for placing the stent by adhesive tape before the procedure. A 4-Fr headhunter catheter was then inserted into the right common carotid artery, and angiographic examination was performed.

**Fig. 3:**
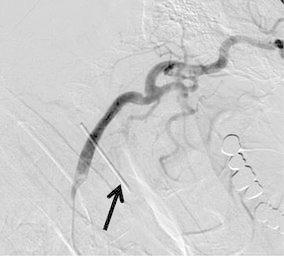
Right carotid angiogram. Radiopaque marker indicating the location of the cutaneous pocket between the origin of the right common carotid artery and the carotid bifurcation is placed on the surface of the patient’s neck (*arrow*)

An angiogram showed a pathologic condition located at the midpoint between the origin of the right common carotid artery and the carotid bifurcation. The radiopaque markers were placed on the neck surface to mark the appropriate position where it was planned to place the stent (Fig. 3).

A covered stent graft (Niti-S stent, Taewoong Medical, Seoul, Korea), with a body diameter of 10 mm and a length of 60 mm, was advanced along the stiff guide wire to the right carotid artery in a similar way reported by previous studies (Chang et al. 2007; Chaloupka et al. 1999).

The stent was deployed to fully cover the artery that the adjacent tumor had invaded and threatened to cause impending rupture, with reference to the body markers. The distal end of the stent was placed below the carotid bifurcation, and the proximal end was placed at the origin of the common carotid artery.

**Fig. 4:**
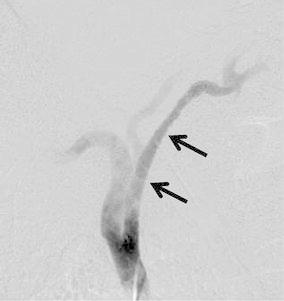
A 60-mm-long, 10-mm-diameter covered stent graft, which protects the artery from direct invasion by the tumor, is deployed in the right carotid artery (*arrows*)

A carotid artery angiogram showed the full expansion and accurate positioning of the stent after stent placement (Fig. 4). The patient was asymptomatic and had no neurological problems related to the procedure. Antiplatelet was not administrated because of his poor general condition.

**Fig. 5:**
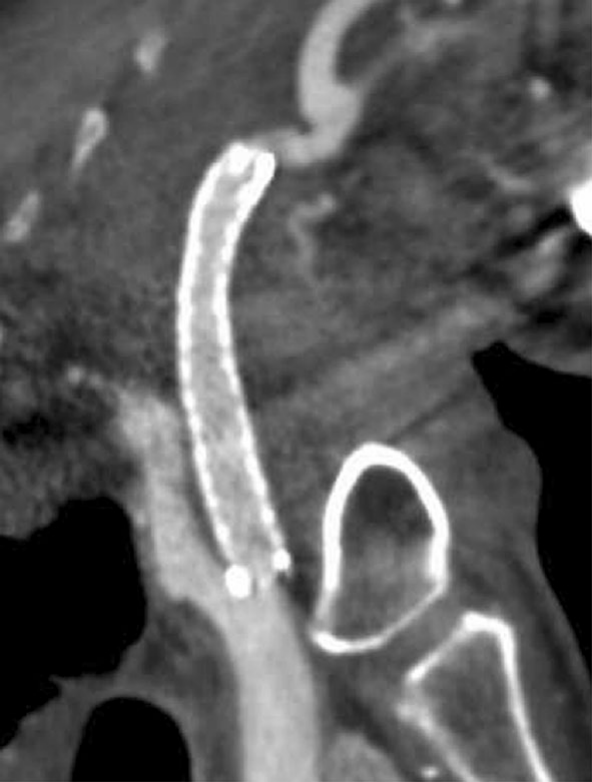
Multiplanar reconstruction contrast-enhanced CT in a sagittal view 2 months after stent placement shows full expansion of the stent and favorable patency of the carotid artery

After 2 months, cervical contrast-enhanced CT revealed that the blood flow through the carotid artery was intact (Fig. 5). Despite subsequent palliative treatment, the patient died 7 months after stent placement due to a respiratory infection. Rupture of the carotid artery did not occur while the patient was alive.

## Discussion

Carotid blowout, or rupture of the carotid artery, is one of the most feared complications of head and neck treatment for malignant disease. Risk factors for CBS include wound infection, radiation therapy, radical resection and stripping of the carotid sheath, flap necrosis with carotid exposure and recurrent tumor (Pyun et al. 2007; Chang et al. 2007, 2008).

Recurrence or regrowth of supraclavicular lymph node metastases is sometimes encountered in patients with advanced head and neck cancer. A metastatic lymph node could directly infiltrate the surrounding structure and lead to the rupture of the carotid artery (Chang et al. 2007).

Especially in patients with advanced cancer, carotid artery rupture is life-threatening. Prompt treatment is required because patients with CBS have a 60 % risk of neurological morbidity and a 40 % risk of mortality (Citardi et al. 1995). Treatment for CBS includes surgery with resection and reconstruction of carotid artery ligation. Recently, endovascular procedures, including embolization with platinum coils and stent-graft placement, have also been useful for the treatment of a ruptured carotid artery. These procedures have many advantages over surgical techniques due to their high success rate, reduced invasiveness, and lower late complication rate (Pyun et al. 2007; Chang et al. 2007, 2008).

Many reports have described the usefulness of covered stent placement and coil embolization in patients with active bleeding due to CBS or impending carotid rupture; however, few reports have described the prophylactic placement of covered stents (Bates and Shamsham 2003). A covered stent-graft could protect the carotid artery from direct tumor infiltration. As a consequence, CBS and formation of a pseudoaneurysm can be prevented.

The current patient presented without episodes of hemorrhage or impending carotid rupture, but the tumor was located in the right supraclavicular fossa and invaded to the adjacent carotid sheath, suggesting impending carotid rupture or formation of a pseudoaneurysm that necessitated emergency treatment. Due to the fact that the general performance status of these patients is often poor, it was assumed that inserting the stent for the purpose of managing the massive bleeding in end-stage patients before the carotid artery were to rupture would be preferable. In addition, once the CBS occurs in these patients, their status is usually worse than at the time of initial chemoradiotherapy. Thus, a vascular intervention is often more invasive to the patient and more technically difficult for the operators.

At first, we considered coil embolization as an alternative endovascular treatment. Coil embolization would require the balloon occlusion test to evaluate the vascular anatomy and contralateral circulation. In addition, it needs many coils and consequently costs very much and takes long time related the procedure. It seemed to be very important to briefly complete the treatment within a minimum session time for avoiding the suffering associated with interventional procedures in a terminal patient. We selected covered stent placement because the covered stent can be easily inserted to the artery and quickly employed. We thought that the covered stent placement could achieve a purpose to prevent carotid blow out in a short time.

In conclusion, although it may not always be necessary to perform stent placement in patients who do not wish for further palliative care, the current case report indicates that the prophylactic placement of a covered stent may be an effective option for the treatment of preventing carotid artery rupture in cases with advanced esophageal cancer; furthermore, it leads to maintaining the patients’ quality of life without any neurologic or stenting sequelae.

## References

[CR1] Bates MC, Shamsham FM (2003). Endovascular management of impending carotid rupture in a patient with advanced head and neck cancer. J Endovasc Ther.

[CR2] Chaloupka JC, Roth TC, Putman CM (1999). Recurrent carotid blowout syndrome: diagnostic and therapeutic challenges in a newly recognized subgroup of patients. AJNR Am J Neuroradiol.

[CR3] Chang FC, Lirng JF, Luo CB (2007). Carotid blowout syndrome in patients with head-and-neck cancers: reconstructive management by self-expandable stent-grafts. AJNR Am J Neuroradiol.

[CR4] Chang FC, Lirng JF, Luo CB (2008). Patients with head and neck cancers and associated postirradiated carotid blowout syndrome: endovascular therapeutic methods and outcomes. J Vasc Surg.

[CR5] Citardi MJ, Chaloupka JC, Son YH (1995). Management of carotid artery rupture by monitored endovascular therapeutic occlusion (1988–1994). Laryngoscope.

[CR6] Katras T, Baltazar U, Colvett K (1999). Radiation-related arterial disease. Am Surg.

[CR7] Meng MB, Zaorsky NG, Jiang C (2013). Radiotherapy and chemotherapy are associated with improved outcomes over surgery and chemotherapy in the management of limited-stage small cell esophageal carcinoma. Radiother Oncol.

[CR8] Pennathur A, Gibson MK, Jobe BA (2013). Oesophageal carcinoma. Lancet.

[CR9] Pyun HW, Lee DH, Yoo HM (2007). Placement of covered stents for carotid blowout in patients with head and neck cancer: follow-up results after rescue treatments. AJNR Am J Neuroradiol.

[CR10] Schoppmann SF, Jesch B, Zacherl J (2013). Lymphangiogenesis and lymphovascular invasion diminishes prognosis in esophageal cancer. Surgery.

[CR11] Schweigert M, Dubecz A, Stein HJ (2013). Oesophageal cancer—an overview. Nat Rev Gastroenterol Hepatol.

